# Comparative whole-genome sequence analysis of *Mycobacterium tuberculosis* isolated from tuberculous meningitis and pulmonary tuberculosis patients

**DOI:** 10.1038/s41598-018-23337-y

**Published:** 2018-03-20

**Authors:** Kiatichai Faksri, Eryu Xia, Rick Twee-Hee Ong, Jun Hao Tan, Ditthawat Nonghanphithak, Nampueng Makhao, Nongnard Thamnongdee, Arirat Thanormchat, Arisa Phurattanakornkul, Somcharn Rattanarangsee, Chate Ratanajaraya, Prapat Suriyaphol, Therdsak Prammananan, Yik-Ying Teo, Angkana Chaiprasert

**Affiliations:** 1 Department of Microbiology Faculty of Medicine, Khon Kaen University, Khon Kaen, 40002 Thailand; 2 Research and Diagnostic Center for Emerging Infectious Diseases (RCEID), Khon Kaen University, Khon Kaen, 40002 Thailand; 3 Saw Swee Hock School of Public Health, National University of Singapore, Singapore, 119077 Singapore; 4 Duke-NUS Medical School, National University of Singapore, Singapore, 119077 Singapore; 5 Drug Resistant Tuberculosis Laboratory, Faculty of Medicine Siriraj Hospital, Mahidol University, Bangkok, 10700 Thailand; 6 Bioinformatics and Data Management for Research Unit, Office for Research and Development, Faculty of Medicine Siriraj Hospital, Mahidol University, Bangkok, 10700 Thailand; 7 National Center for Genetic Engineering and Biotechnology, National Science and Technology Development Agency, Ministry of Science and Technology, Pathum Thani, 12120 Thailand; 8 NUS Graduate School for Integrative Sciences and Engineering, National University of Singapore, Singapore, 119077 Singapore; 9 Genome Institute of Singapore, Singapore, 138672 Singapore; 10 Department of Statistics and Applied Probability, National University of Singapore, Singapore, 119077 Singapore; 11 Life Sciences Institute, National University of Singapore, Singapore, 119077 Singapore

## Abstract

Tuberculous meningitis (TBM) is a severe form of tuberculosis with a high mortality rate. The factors associated with TBM pathogenesis are still unclear. Using comparative whole-genome sequence analysis we compared *Mycobacterium tuberculosis* (*Mtb*) isolates from cerebrospinal fluid of TBM cases (n = 73) with those from sputum of pulmonary tuberculosis (PulTB) patients (n = 220) from Thailand. The aim of this study was to seek genetic variants of *Mtb* associated with TBM. Regardless of *Mtb* lineage, we found 242 variants that were common to all TBM isolates. Among these variants, 28 were missense SNPs occurring mainly in the *pks* genes (involving polyketide synthesis) and the PE/PPE gene. Six lineage-independent SNPs were commonly found in TBM isolates, two of which were missense SNPs in *Rv0532* (*PE_PGRS6*). Structural variant analysis revealed that PulTB isolates had 14 genomic regions containing 2–3-fold greater read depth, indicating higher copy number variants and half of these genes belonged to the PE/PPE gene family. Phylogenetic analysis revealed only two small clusters of TBM clonal isolates without support from epidemiological data. This study reported genetic variants of *Mtb* commonly found in TBM patients compared to PulTB patients. Variants associated with TBM disease warrant further investigation.

## Introduction

Tuberculosis (TB), a major infectious disease caused by *Mycobacterium tuberculosis* (*Mtb*), accounts for 1.8 million deaths and 10.4 million new cases annually^1^. Extrapulmonary TB accounts for 15% of all TB cases^1^. Tuberculous meningitis (TBM) is a severe form of extrapulmonary TB affecting the central nervous system (CNS) and accounts for 5% of all extrapulmonary TB cases^2^. Despite proper treatment, the mortality rate of TBM ranges from 25% in HIV-negative patients^3^ up to 70% in HIV-positive cases^4^.

Not all pulmonary TB patients develop TBM and it is possible to develop TBM without pulmonary TB. TBM usually begins with respiratory infection followed by haematogenous dissemination to the CNS^5^. Pathogenesis of TBM occurs when subependymal or subpial tubercles (Rich foci), seeded during bacteremia of disseminated disease or primary infection, rupture into the subarachnoid space^6^. However, the mechanisms by which *Mtb* leaves the lung, enters the brain through the blood-brain barrier and causes the subsequent cerebral pathology, remain unclear.

TBM is commonly found in young people, especially those with primary TB, but can also occur in immune-compromised older individuals, especially those with HIV infection^7^. Host genetic variation of immunological recognition molecules, such as TIRAP^8^ and TLR2^9^ that are associated with the innate immune response controlling the dissemination of the pathogens, were found to be associated with TBM susceptibility. Genetic factors of *Mtb* associated with the development of TBM remain poorly known. The East-Asian/Beijing lineages of *Mtb* are more commonly associated with the development of TBM than is the Euro-American lineage^10,11^. However, the genetic factors, defined by molecular typing, that are used to classify the lineages of *Mtb* do not provide a clear genetic determinant associated with TBM. Several genes (*Rv0311*, *Rv0805*, *Rv0931c*, *Rv0986*, and *MT3280*) have been reported to influence the invasion or survival of *Mtb* in the CNS but not in the lung tissue^12^. *Rv0931c* (*pknD*) encoding a serine/threonine protein kinase plays an important role in brain endothelial cell adhesion and invasion, hence enabling *Mtb* to cross the blood-brain barrier in TBM^13^. These genes can be found in all lineages of *Mtb*, suggesting that any study of genetic factors should be lineage-independent. Furthermore, additional genes involving TBM pathogenesis might remain to be discovered. Therefore, high-resolution methods such as whole-genome sequencing (WGS) of *Mtb* isolates causing TBM should provide a new insight into genetics of *Mtb* and mechanisms associated with TBM pathogenesis.

High-throughput sequencing analysis provides insights into mycobacterial genetics related to pathogenesis, diagnosis, epidemiology and treatment of TB. However, there have been few WGS analyses of *Mtb* causing TBM. The one previous such study used only eight isolates causing TBM and did not take into account the lineage of *Mtb*^14^.

In this study, 293 *Mtb* isolates from Thai TB patients, including 73 from TBM cases and 220 isolates from PulTB cases, were investigated using comparative WGS analysis adjusted for lineage to investigate any genetic variant of *Mtb* that might be associated with causing TBM.

## Results

### Characteristics of TBM and PulTB cases

Two hundred and ninety-three TB patients, including 73 TBM and 220 PulTB cases, were recruited in this study. The average age of TBM cases (34.51 years) was significantly lower than for PulTB patients (41.72 years) (p = 0.0009). Most patients in both groups (64–69%) were male (Table 1). About half of the *Mtb* isolates from TBM cases (48%) were pan-susceptible whereas most from PulTB (90%) were resistant to at least one anti-TB drug (Table 1).

**Table 1:** Characteristics of *M. tuberculosis* isolates from TBM and PulTB patients.

Characteristics	TBM cases (n = 73)	PulTB cases (n = 220)
Age, mean (+− SD)	34.51 (+−14.48)^a^	41.72 (+−13.95)^b^
Male Gender, % (proportion)	64.38 (47/73)	68.84 (148/215)^b^
Year of collection	1998–2007^d^	2003–2013^c, e^
**Drug resistance pattern (number (%))**		
Pan-susceptible	35 (47.95)	17 (7.73)
I resistant	5 (6.85)	0 (0)
IS resistant	2 (2.74)	0 (0)
S resistant	1 (1.37)	0 (0)
MDR	3 (4.11)	93 (42.27)
QDR	1 (2.08)	82 (37.27)
XDR	0 (0)	23 (10.45)
No DST results	26 (35.62)	5 (2.27)

Note: ^a^Age data for 18 TBM patients were missing. ^b^Age data for 27, ^c^gender data for 5 and year of collection data for 1 pulmonary TB patients were missing, ^d^74% (54/73) of isolates from TBM cases were collected during 2004–2007, ^e^31% (68/219) of isolates from PulTB cases were collected during 2004–2007. I: isoniazid, S: streptomycin, MDR: multidrug resistance, QDR: quinolone drug resistant (MDR with resistant to fluoroquinolone), XDR: extensively drug resistant, DST: drug susceptibility test.

### Lineage distribution between TBM and PulTB cases

Distribution of *Mtb* lineages differed significantly between TBM and PulTB cases (p < 0.0001). The East-Asian lineage predominated in both disease types but the proportion of isolates of the Indo-Oceanic lineage was significantly higher among TBM patients (p < 0.0001). The proportions of sublineages belonging to the East-Asian and the Euro-American lineage were not significantly different between TBM and PulTB (Table 2).

**Table 2:** Distribution of lineages based on RD (LSP) markers of *M. tuberculosis* (*Mtb*) isolated from TBM and PulTB patients.

Lineages and sublineages of *Mtb*	TBM cases (n = 73)	PulTB cases (n = 220)
East-Asian (number (%))	37 (50.68)	173 (78.63)
Sublineage 2.1	0	18
Sublineage 2.2.1	32	134
Sublineage 2.2.2	0	9
Sublineage 2.2.1.1	5	9
Sublineage 2.2.1.2	0	1
Sublineage with RD105,142,150,181 and 207 deletion	0	1
Sublineage with RD105 and 181 deletion	0	1
**Euro-American (number (%))**	6 (8.22)	17 (7.73)
Sublineage 4.3.4 and others	0	1
Sublineage 4.5	1	3
Sublineage 4.8	2	1
Sublineage 4.2, 4.4, 4.7, H37Rv-like and others	3	12
**Indo-Oceanic (number (%))**	30 (41.10)	29 (13.18)
Ancestral lineage* (number (%))	0	1 (0.45)

Note: Ancestral lineage* refers to Strain without any deletion of 31 RDs based on RD-analyzer.

### Phylogeny of *Mtb* isolates from cerebrospinal fluid of TBM cases and sputum of PulTB patients

**Figure 1:**
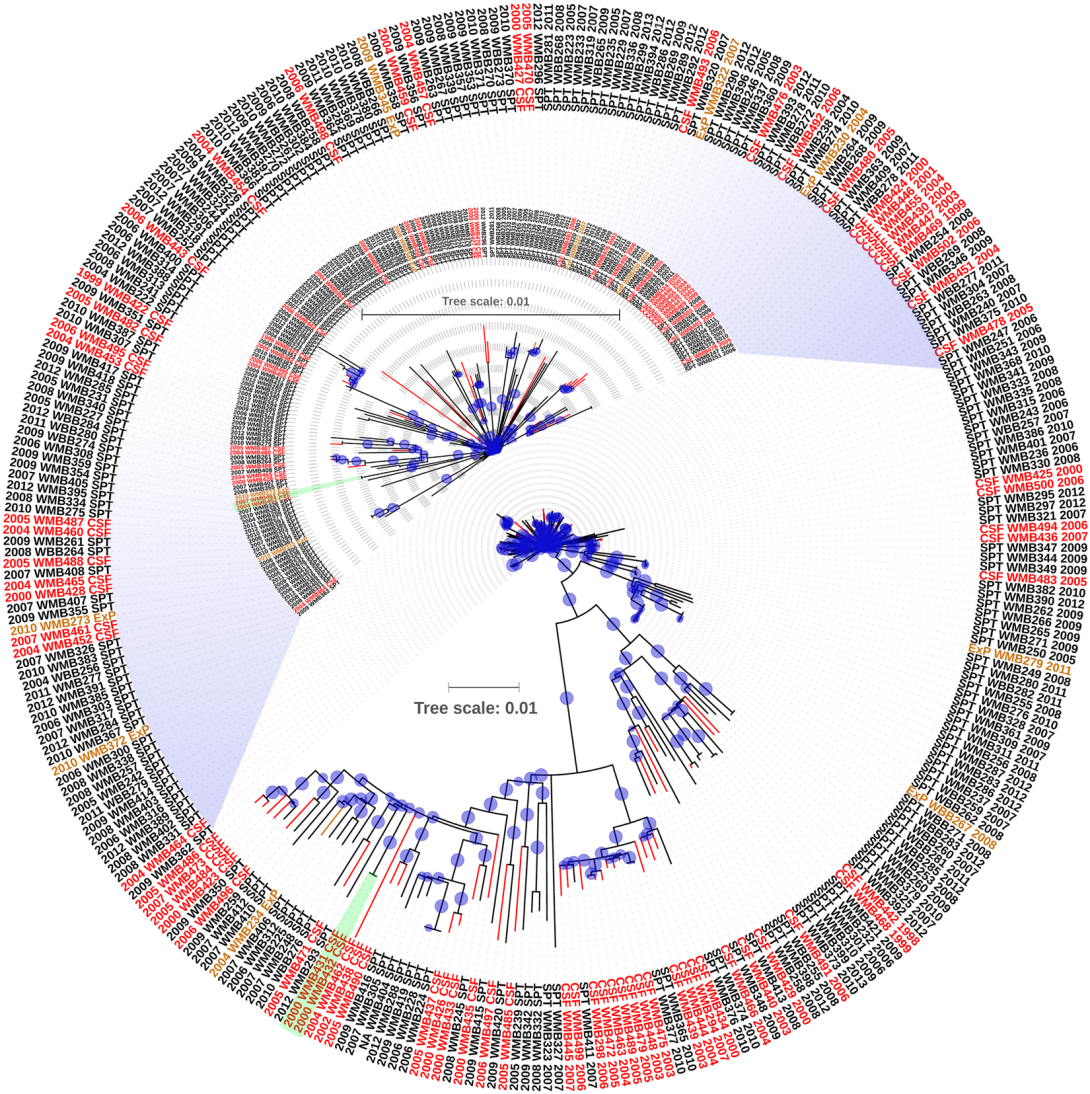
Phylogenetic analysis of *M. tuberculosis* isolates from TBM and PulTB patients. The phylogenetic tree was inferred using the maximum likelihood method with general time reversible and gamma distribution model using 4,490 high-confidence SNPs and compared to the H37Rv reference genome. The bootstrap consensus tree was inferred from 1,000 replicates. Blue circles refer to bootstrap values and the size of each circle is proportional to its value (most of the bootstrap values are 100). Black, red and orange branches and letters refer to *Mtb* isolates from sputum (SPT) of PulTB cases, cerebrospinal fluid (CSF) of TBM cases, and extrapulmonary (ExP) samples other than CSF, respectively. The upper portion of the tree has been magnified as an inset. Two small clusters of *Mtb* isolated from TBM patients are indicated in green boxes. Numbers in the outer ring (e.g. 2009) refer to year of collection and NA indicates unavailable data.

A phylogenetic tree based on 4,490 high-confidence SNPs of 293 *Mtb* isolates (73 TBM and 220 PulTB) showed that *Mtb* isolates from both disease types were scattered throughout the tree. The largest clonal cluster of 4 isolates from PulTB (SPT WMB256, 283, 286 and 287) and 2 two-isolate clonal clusters from TBM (CSF WMB452 and 461 and CSF WMB431 and 432) are shown (Fig. 1). Only the cluster of CSF WMB 431 and 432 were isolated in the same year but from different patients.

### SNPs and small indels common to all *Mtb* isolates from TBM cases but not found in all PulTB isolates

**Figure 2:**
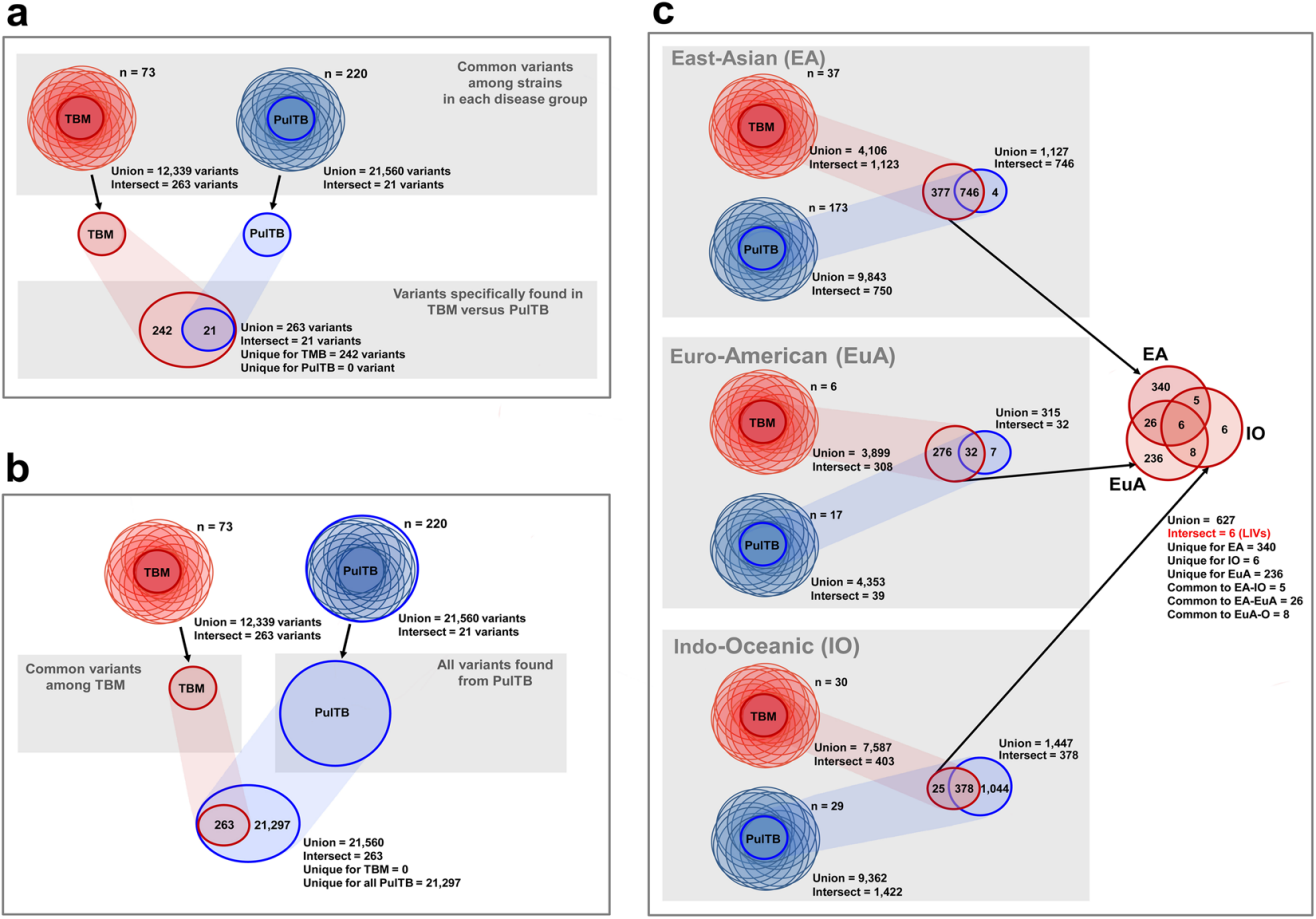
Venn diagrams illustrating the variant comparison between *M. tuberculosis* isolates from TBM and PulTB. (**a**) Comparison of variants between TBM and PulTB. (**b**) Comparison between variants common to all TBM isolates and all variants from PulTB. (**c**) Comparison of variants common to all lineages of *Mtb* isolated from TBM patients.

Two hundred and forty-two variants were common to all isolates from TBM patients, but were not represented among the variants common to all PulTB isolates (Fig. 2a). Of these, 28 variants were missense SNPs (Table 3). However, the variants common to all TBM isolates all occurred in some proportion of the PulTB isolates (Fig. 2b). To analyze the lineage-independent variants (LIVs) specific to TBM, the comparisons were done separately for each lineage. Six LIVs were noted from TBM isolates (i.e. they occurred only in isolates from TBM cases and in all three major lineages) (Fig. 2c). Two of the LIVs (623,472 A > G and 623,508 C > G) were missense SNPs within *Rv0532* (*PE_PGRS6*) (Table 3). Gene function is unknown for most genes containing missense SNPs and LIVs common to all TBM isolates (Supplementary Table S1). Indo-Oceanic lineage isolates from TBM patients had fewer lineage-specific variants (25 variants) than those from the East-Asian (377 variants) and Euro-American lineages (276 variants) (Fig. 2c).

**Table 3:** Characteristics of 242 variants common to all TBM isolates but found in varying proportions among PulTB isolates.

Variant information	Number of variants	Notes
Types		
- SNPs	240	
- Indels (small insertions)	2	
**Impact and region**		
- Missense SNPs	28	2 LIVs
- Silent (synonymous) SNPs	16	
- Downstream gene variants	15	1 insertion
- Upstream gene variants	52	2 LIVs
- Intergenic region variants	131	2 LIVs, 1 insertion

Note: LIV = lineage-independent variant.

### Structural variants specifically found in *Mtb* isolates from TBM cases compared with those from PulTB patients

**Figure 3:**
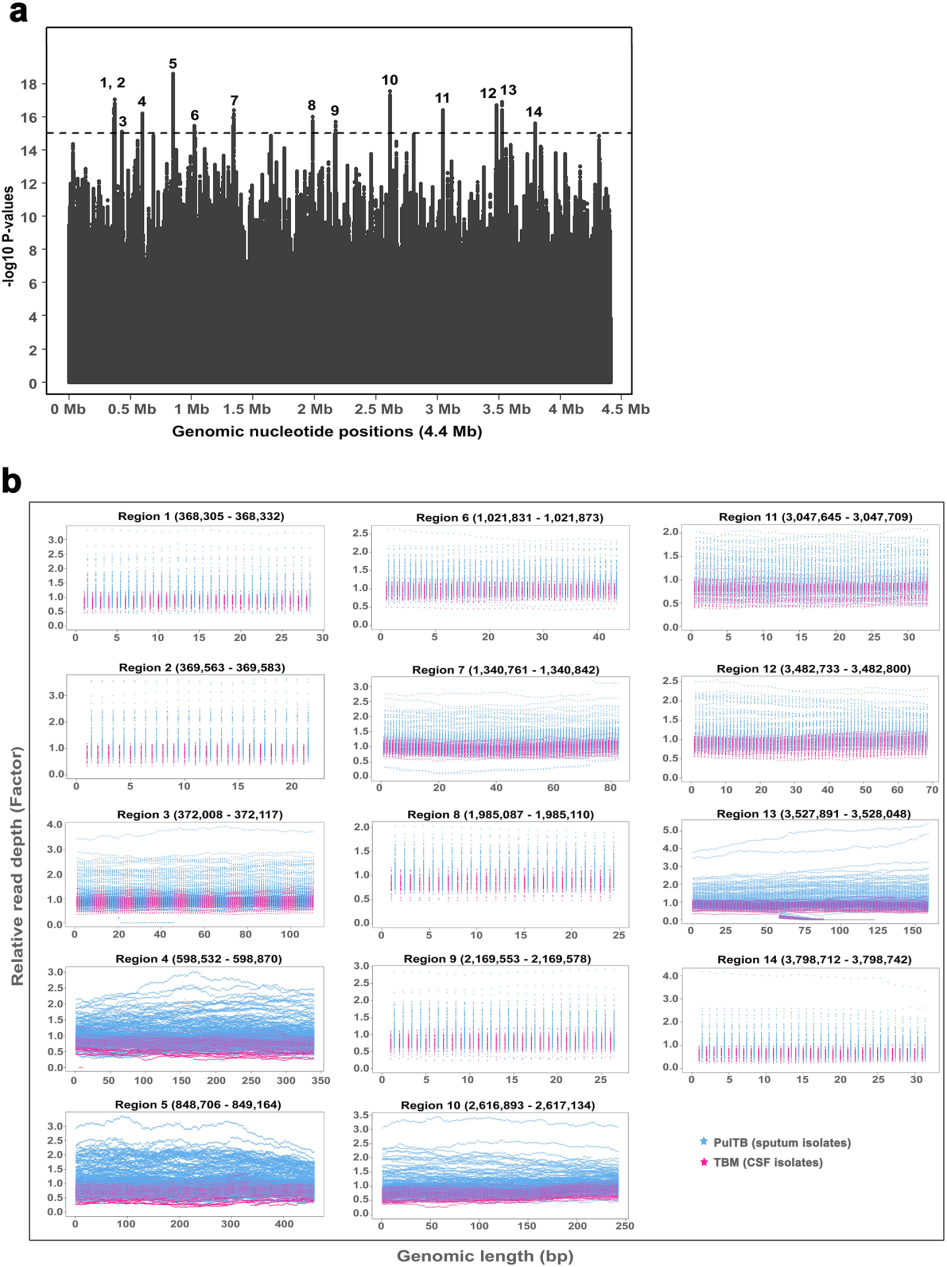
Structural variant comparison across genomic nucleotide positions between *M. tuberculosis* isolates from TBM and PulTB. (**a**) 14 genomic regions differ between *Mtb* isolates from TBM and PulTB patients based on –log10 p-values above 15. (**b**) Relative depth coverage of *Mtb* isolates from PulTB patients was 2–3 fold higher than TBM isolates, indicating higher copy number variants found in PulTB isolates. Affected genes and their functions are described in Additional file 2: Table S2. Relative read-depth (factor) refers to the ratio between the read depth at a particular nucleotide position and average read depth across all genomic nucleotide positions.

Because our WGS analysis pipeline identified only small indels, Wham^15^, a recently developed analysis tool for structural variants (SVs) covering large indels and other SV types, was used. Wham revealed 86,249 structural variants (large deletions, large insertions, duplications and inversions) among the 73 isolates from TBM cases and 198,104 variants among 220 PulTB isolates. However, no group-specific common structural variant was found (no figure shown). Large-indel analysis using RD-Analyzer showed that PulTB isolates had 14 genomic regions with 2–3 fold higher relative read depths indicating variation in copy number between PulTB and TBM isolates (Fig. 3a,b). The functions of the most of affected genes were unknown but half belonged to the PE/PPE gene family (Supplementary Table S2).

## Discussion

We analyzed the WGS of 73 *Mtb* isolates from TBM patients and 220 *Mtb* isolates from PulTB patients in Thailand. To investigate the genetic variants of *Mtb* causing TBM, comparisons of variants between the two disease types were performed.

Two hundred and forty-two SNP/small indel variants were found in all *Mtb* isolates from TBM cases but were not commonly found in those from PulTB patients. Among these were 28 missense SNPs of 26 genes that included 3 PPE/PE-family genes and 2 *pks* genes encoding polyketide synthase. It has been suggested that the PE/PPE gene family encodes virulence factors and are a possible source of antigenic variation influencing immune evasion^16^. Whether or not these genes have roles in TBM pathogenesis by interacting with the host immune system requires further investigation.

TB pathogenesis is often regarded as a function of *Mtb* lineage^11,17,18^. The East-Asian lineage has been suggested as the most virulent and the Euro-American lineage as the most benign in relation to frequency and severity of TBM^11^. We found a predomination of the East-Asian lineage in both disease types, but a lower proportion of the Indo-Oceanic lineage in PulTB cases (13.2% vs 41.1%). This might be due to the random sampling process that skewed the lineage distribution of the PulTB strains: the larger sample set from our previous study included around 30% of Indo-Oceanic lineage isolates among PulTB strains^19^. In the Euro-American lineage, the presence of *pks15/1* has been suggested as the relevant genetic determinant. This polyketide synthase gene encodes a phenolic glycolipid (PGL) that inhibits the release of pro-inflammatory cytokines in a mouse model^20^. However, *pks15/1* was also found in other lineages of *Mtb* that cause TBM (Indo-Oceanic and East-African-Indian lineages). Therefore, lineage affiliation does not fully explain TBM pathogenesis. We further analyzed LIVs by calling the variant set common to isolates of all three lineages of *Mtb* from TBM patients relative to PulTB isolates. Of the 6 LIVs found, 2 were missense SNPs in *Rv0532 (PE_PGRS6)*. These variants were associated with TBM regardless of lineage of *Mtb*. The function of this gene, in the PE gene family, remains unknown. Functional analysis of this gene in relation to TBM pathogenesis is, therefore, warranted.

In addition, isolates of the Indo-Oceanic lineage causing TBM had markedly fewer variants than did other lineages indicating more genetic diversity of Indo-Oceanic lineage causing PulTB but more genetically conserved for those causing TBM. The Indo-Oceanic and the East-Asian lineages of *Mtb* are known to induce higher concentrations of pro-inflammatory cytokines than does the Euro-American lineage^17^. The Indo-Oceanic lineage of *Mtb* causing TBM may, therefore, have distinct genetic properties. However, this difference might be just the consequence of the different proportions of this lineage between TBM and PulTB patients.

Although 242 variants (and 6 LIVs) were common to all TBM isolates, they also occurred with varying frequency among PulTB isolates. We hypothesize that particular strains of *Mtb* with these 242 SNPs/ small indels (or 6 LIVs) tended to cause TBM. Some *Mtb* causing PulTB also contain these 242 variants (or 6 LIVs). We hypothesize that these strains have the potential to cause TBM in susceptible hosts but that the hosts from which they were recovered were not susceptible individuals. Interactions between host and pathogen must be involved in the pathogenesis of TBM: we have identified the potential genetic variants on the pathogen side.

Based on the analysis pipeline using standard tools including Samtools and GATK, small indels were identified. The SV caller, Wham^15^, was used to identify large indels and structural variants (SVs). Several SVs were identified among TBM and PulTB isolates. However, unlike small variants, no SV was common to all isolates of a particular disease type. As the objective of our study was to identify specific genetic variants common to *Mtb* isolates causing TBM, we did not further analyze these SVs. We further used RD-Analyzer^21^ to call the genetic region variants associated with TBM and identified at least 14 regions in which PulTB isolates had 2–3 fold higher relative read depths than did TBM isolates. Interestingly, half of these 14 regions belonged to the PE/PPE gene family. Therefore, the results of SV analysis support the results obtained from the SNPs. The PE/PPE gene family may be associated with virulence factors and antigenic variation influencing immune evasion^16^: copy number of these appears to differ between PulTB and TBM isolates, perhaps influencing the different pathogenesis mechanism of TBM. However, we emphasize that functions are unknown for the majority of genes found to differ between TBM and PulTB. Functional analysis of these genes is a priority for future research.

Phylogenetic analysis of *Mtb* isolates from TBM and PulTB patients showed that there is no specific clade of isolates associated with TBM. Transmission of TBM-causing clones between patients seems rare: only two small clonal clusters (2 isolates each) were found. The first cluster (WMB452 and WMB461) of TBM cases was not supported by the epidemiological information (year of collection and home province of the patients). Although lacking known contact history, the second cluster (WBM431 and WMB432) was isolated in the same year from different patients (47-year-male and 83-year-female but no home province information was available). Therefore, we cannot exclude the possibility that transmission could occur. The discussion of cluster analysis for PulTB group was not included, as such information will be reported in a separate study.

Previously, *Rv0931c* (*pknD*), *Rv0311*, *Rv0805*, *Rv0931c*, *Rv0986*, and *MT3280* were reported as important genes enabling the bacteria to cross the blood–brain barrier in TBM^12,13^. However, in our study we could not find common variants affecting these genes. A previous WGS study of TBM isolates investigated only a few isolates of TBM and did not take into account the lineages of *Mtb*^14^. They found variants of *Rv0311* and *Rv0619* in all eight of their TBM isolates but not in the sputum control isolates. None of these variants was shared by all eight strains but 36 variants involving 10 genes (*PE-PGRS10, PPE58, PE_PGRS49, lppD, PE_PGRS21, Rv0278c, embR, PE_PGRS19, PPE53* and *PPE24*) were each found at least half of the strains. In our more extensive study from a different geographical region, we found 242 variants common to all 73 TBM isolates, none of which belonged to genes reported in the previous study. Possibly *Mtb* causing TBM in Thailand is more genetically conserved than strains used in previous studies. Nevertheless, our results partly supported the previous study in pointing to an association between variants of the PE/PPE gene family of *Mtb* and TBM. However, the gene variants detected from the previous study are not the same set as found in our study.

In general, rates of drug resistance (any drug resistance) do not differ significantly (around 25–30%) between TBM and PulTB isolates^22^, including those from Thailand^23,24^. A recent study from China noted a high rate (48%) of drug-resistant TBM isolates, 80% (20/25 isolates) of which had the Beijing (East-Asian) genotype^25^. Previously, we reported 25% of isolates were drug resistant and 50% of isolates exhibited the Beijing genotype among TBM cases in Thailand during 1998–2007^10^. The different proportions of the Beijing genotype in the studies from China and Thailand might have led to different rates of drug resistant TBM being found. Here, the TBM strains recruited from the previous study^10^ were selected to include around 20% of drug resistant isolates and 50% of the Beijing genotype. Among the PulTB strains selected for inclusion, around 90% were M/XDR-TB: these strains were selected to study genetics related to drug resistance (not reported in this study) and were also used as a control group for the genome analysis of TBM strains. Although there is the background difference of *Mtb* between the two studied groups, especially with respect to drug susceptibility patterns and lineage distribution, the genetic analysis of TBM strains adjusted for these factors, thus excluding their confounding effects. The analysis was performed by comparing the common variant set that was called among heterogeneous drug resistant phenotypes (including both drug-susceptible and drug-resistant strains).

Possibly, drug resistance-associated mutations could alter the pathogen’s fitness and its ability to cross the blood-brain barrier causing TBM^26^. Such mutations might be traced based on the absence of specific mutations in all TBM strains, or shared mutations found in all PulTB controls. Unfortunately, we had too few drug-resistant TBM strains in our study to identify SNPs that may be associated with such a phenomenon. Furthermore, we found no common mutation shared by all drug-resistant PulTB strains. In the future, strains with particular SNPs associated with drug resistance could be experimentally tested for their ability to cause TBM.

It seems that analysis of an increased number of strains leads to discovery of a smaller set of common variants. However, our study provides the largest collection of sequences from TBM isolates to date. Furthermore, variants associated with TBM were identified in a lineage-independent fashion. Our focus was on the genetic variants specifically found in *Mtb* isolates causing TBM compared to pulmonary TB. We analyzed rather few isolates from other types of extrapulmonary TB. This is a clear limitation of our study. However, eight additional non-TBM extrapulmonary strains (3 cutaneous, 1 lymph node, 1 pleural, and 3 nonspecific tissues) all showed the 242 SNPs commonly found in TBM strains (data not shown). Analysis of a larger number of non-TBM extrapulmonary strains might identify a smaller set of variants that are universal among all such strains.

In summary, our study showed that *Mtb* isolates from TBM patients had genetic variants likely associated with TBM pathogenesis. Based on both SNP and SV analysis, several PE/PPE genes were identified as associated with TBM. The function of most of these genes was unknown. Missense SNPs of *Rv0532* (*PE_PGRS6*), which are lineage-independent variants, are among the most promising genetic variants associated with TBM pathogenesis.

## Methods

### *Mtb* isolates and setting

In total, 293 *Mtb* isolates were retrieved from stock cultures of clinical isolates deposited at the Drug-Resistant Tuberculosis Research Fund, Faculty of Medicine Siriraj Hospital, Mahidol University, Bangkok, Thailand. Included were 220 *Mtb* isolates from sputum (SPT) of PulTB patients collected from 1998 to 2007 and 73 *Mtb* isolates from cerebrospinal fluid (CSF) of TBM patients collected from 2003 to 2013. Many of the TBM isolates were a subset of those used in our previous study^10^. The subset was selected to have the same proportions of drug resistance status and genotypes as the full set. PulTB isolates, selected from a collection of drug resistant isolates (n = 198) and pan-drug susceptible isolates (n = 17), were used as a control group. The study protocol was approved by the Ethical and Scientific Committees of the Faculty of Medicine Siriraj Hospital, Mahidol University (ECNo. Si 029/2557). All experiments dealing with viable pathogens were done in a standard biosafety level 2 laboratory with highly regulated experimental protocol, waste management and personal protective equipment (BSL2 plus). All methods were performed in accordance with the relevant guidelines and regulations. This study used left-over specimens without the information that could lead to identification of any study participant and no informed consent is required.

### Culture of *Mtb* and extraction of genomic DNA

All *Mtb* isolates were sub-cultured onto Löwenstein–Jensen media and incubated at 37 °C for four weeks. Genomic DNA was extracted from multiple loopfuls of *Mtb* colonies using the cetyl-trimethyl-ammonium bromide-sodium chloride method^27^.

### Drug susceptibility test

Phenotypic drug susceptibility tests for anti-TB drugs were performed using standard proportional methods^28^ on Middlebrook 7H10 agar plates. Drug concentrations used were 0.2 mg/l for isoniazid, 1.0 mg/l for rifampicin, 5.0 mg/l for ethambutol and ethionamide, 6.0 mg/l for amikacin and kanamycin, and 2.0 mg/l for streptomycin, *p*-aminosalicylic acid, ofloxacin, levofloxacin, moxifloxacin and gatifloxacin. *Mycobacterium tuberculosis* H37Rv was used as susceptible reference strain.

### Whole-genome sequencing

Sequencing of the *Mtb* isolates was performed at the Genome Institute of Singapore, Singapore. Genomic libraries were prepared according to the recommendations of the TrueSeq DNA sample preparation kit (Illumina, San Diego, CA) for the MiSeq platform (Illumina) generating 250-bp read lengths or NEBnext Ultra kit (Illumina, San Diego, CA) for Hiseq (Illumina) platform generating 150-bp read lengths. The sequence data have been deposited in the Sequence Read Archive (SRA) containing 293 biosample accession Nos. SAMN07236248 – 540 under the bioproject accession No. PRJNA390471.

### Bioinformatics and data analysis

#### Mapping of sequencing reads

The overall quality of sequence read was checked using FastQC version 0.11.3^29^. All sequences with an average quality score above 36 were retained. Reads shorter than 36 bp and possibly contaminating adaptor sequences were excluded using Trimmomatic version 0.33^30^. Paired-end raw reads of each isolate were mapped to the *Mtb* H37Rv reference genome (GenBank accession number: NC_000962.3) using BWA MEM version 0.7.12^31^. Samtools version 0.1.19^32^ was used for SAM-BAM format conversion and sorting of mapped sequences. Local realignment of the mapped reads was performed using GATK version 3.4.0^33^. The stat reports were generated using GATK and Samtools, indicating that the average depth coverage of the mapped sequences was 118.88 ± 69.62 (141.12 ± 73.84 for TBM and 111.47 ± 66.70 for PulTB) and the average mapping rate of the sequences was 97.79% ± 1.65% (97.70 ± 0.43% for TBM and 97.82 ± 1.89% for PulTB).

#### SNPs and small-indel analysis of Mtb isolated from TBM and PulTB patients

Variants, including single nucleotide polymorphisms (SNPs) and small indels, were called using GATK and Samtool tools^32^. Variant sites were filtered based on the following criteria: mapping quality >50 (-C in Samtools calling), base quality/base alignment quality >20 (-Q in Samtools calling), >10 reads or ≤2,000 reads (-d in Samtools filter) covering each site. To maximize specificity, the called variants were selected from the intersection of those identified by Samtools and GATK. The snpEff version 4.1^34^ was used for variant annotation. Additionally, heterozygous SNPs with allelic frequencies of <75% or read-depth <10 reads were excluded. Those remaining and satisfying all the above criteria were regarded as high-confidence variants.

The variants were merged among all isolates in each group (TBM versus PulTB) using GATK^33^. The intersection of variants (common variants) for each disease group were compared. To exclude the variants associated with the lineages of *Mtb*, the intersection of variants from each lineage (East-Asian, Indo-Oceanic and Euro-American lineages) were separately called and then analyzed and then compared between the two disease groups. The common variants from TBM isolates were also compared to all identified variants called from PulTB isolates.

#### Structural variant analysis of Mtb isolated from TBM and PulTB patients

Structural variants (deletions, insertions, inversions and duplications) were analyzed using Wham^15^. Sorted BAM files with realignment were used based on the default parameters of Wham. The variants among all isolates from each disease group were merged and then the intersection of variants (common variants) for each disease group were compared using in-house python script.

RD (region of difference) analysis was done using RD-Analyzer covering 31 known RDs for *Mtb* lineage classification^21^. Novel RD analysis was performed based on the analysis of read count through genomic positional comparison across 4,411,532 bp between *Mtb* isolates from TBM and PulTB patients. The depth coverage of each nucleotide position proportional to the average depth coverage of all positions (relative read depth or factor) of each genome was analyzed. The average relative read depth of each genomic position between *Mtb* isolates from PulTB versus those from TBM cases were compared based on t-tests. Genomic regions with continuous nucleotide positions with -log10 p-values larger than 15 were called as candidate genomic region variants specific to TBM isolates.

#### Phylogenetic analysis

Phylogenetic analysis of the 4,490 high-confidence SNPs identified among 293 *Mtb* isolates (73 TBM and 220 PulTB) was performed based on the maximum likelihood (ML) method using MEGA-6^35^ with a general time-reversible (GTR) model of nucleotide substitution and a gamma model of rate heterogeneity. The phylogenetic tree was constructed based on 1,000 bootstrap replicates. Visualization of the phylogenetic tree was performed using iTOL^36^.

### Data analysis

Descriptive statistics were used to describe the characteristics of the *Mtb* isolates. The comparison of average age of the patients between TBM and PulTB groups was performed using the independent t-test. The comparisons of gender proportions and drug susceptibility proportions between the two disease types were performed using chi-square tests. SPSS version 16 (SPSS Inc., Illinois, USA) was used. For all analyses, a p-value <0.05 was considered to be statistically significant.

### Data availability statement

The datasets generated during and/or analysed during the current study are available in the Sequence Read Archive (SRA) containing 293 biosample accession Nos. SAMN07236248 – 540 under the bioproject accession No. PRJNA390471.

## Electronic supplementary material

Supplementary Tables
