# Supplementary material for: Comparative whole-genome sequence analysis of Mycobacterium tuberculosis isolated from tuberculous meningitis and pulmonary tuberculosis patients
Source: Sci Rep. 2018 Mar 20;8:4910. doi: 10.1038/s41598-018-23337-y (PMC5861094; doi:10.1038/s41598-018-23337-y)
Supplement: Supplementary file 1 — Supplementary Tables [file 41598_2018_23337_MOESM1_ESM.pdf]

Comparative whole-genome sequence analysis of *Mycobacterium tuberculosis* isolated from tuberculous meningitis and pulmonary tuberculosis patients

Kiatichai Faksri[1,2,*], Eryu Xia[3], Rick Twee-Hee Ong[3], Jun Hao Tan[4], Ditthawat Nonghanphithak[1,2], Nampueng Makhao[5], Nongnard Thamnongdee[5], Arirat Thanormchat[5], Arisa Phurattanakornkul[5], Somcharn Rattanarangsee[5], Chate Ratanajaraya[5], Prapat Suriyaphol[6], Therdsak Prammananan[7], Yik-Ying Teo[3,8,9,10,11], Angkana Chaiprasert[5*]

[1]Department of Microbiology Faculty of Medicine, Khon Kaen University, Khon Kaen 40002, Thailand.
[2]Research and Diagnostic Center for Emerging Infectious Diseases (RCEID), Khon Kaen University, Khon Kaen 40002, Thailand.
[3]Saw Swee Hock School of Public Health, National University of Singapore 119077, Singapore.
[4]Duke-NUS Medical School, National University of Singapore 119077, Singapore.
[5] Drug Resistant Tuberculosis Laboratory, Faculty of Medicine Siriraj Hospital, Mahidol University, Bangkok 10700, Thailand.
[6]Bioinformatics and Data Management for Research Unit, Office for Research and Development, Faculty of Medicine Siriraj Hospital, Mahidol University, Bangkok 10700, Thailand.
[7]National Center for Genetic Engineering and Biotechnology, National Science and Technology Development Agency, Ministry of Science and Technology, Pathum Thani 12120, Thailand.
[8]NUS Graduate School for Integrative Sciences and Engineering, National University of Singapore 119077, Singapore.
[9]Genome Institute of Singapore 138672, Singapore.
[10]Department of Statistics and Applied Probability, National University of Singapore 119077, Singapore.
[11]Life Sciences Institute, National University of Singapore 119077, Singapore.

Correspondence and requests for materials should be addressed to K.F (email: kiatichai@kku.ac.th) and A.C (email: angkana.cha@mahidol.ac.th)

Positions (SNPs)	Genes	Gene function	Note
**Common missense SNPs specifically found in *Mtb* causing TBM compared to PulTB**			
12,210 (A>G)	*Rv0103c (ctpB)*	Cation-transporting ATPase	
225,323 (T>C)	*Rv0193c*	Unknown	
475,178 (T>C)	*Rv0395*	Unknown	
502,589 (C>G)	*Rv0417 (thiG)*	Thiamine biosynthesis	
598,475 (G>A)	*Rv0507 (mmpL2)*	Unknown (Fatty acid transport)	
623,472 (A>G)	*Rv0532 (PE_PGRS6)*	Unknown	LIV
623,508 (C>G)	*Rv0532 (PE_PGRS6)*	Unknown	LIV
648,002 (T>G)	*Rv0556*	Unknown	
775,639 (T>C)	*Rv0676c (mmpL5)*	Unknown (Fatty acid transport)	
1,552,547 (G>A)	*Rv1378c (Rv1378c)*	Unknown	
1,885,772 (G>A)	*Rv1662 (pks8)*	Polyketide synthesis	
1,944,402 (T>C)	*Rv1716*	Unknown	
2,057,774 (A>T)	*Rv1815*	Unknown	
2,143,328 (G>C)	*Rv1895*	Unknown (cellular metabolism)	
2,269,780 (T>C)	*Rv2024c*	Unknown	
2,270,102 (A>G)	*Rv2024c*	Unknown	
2,362,041 (C>A)	*Rv2101 (helZ)*	Has helicase activity	
2,386,389 (G>A)	*Rv2125 (Rv2125)*	Unknown	
2,415,656 (G>C)	*Rv2155c (murD)*	Peptidoglycan biosynthesis	
2,910,461 (G>T)	*Rv2584c (apt)*	Purine salvage	
2,911,293 (C>G)	*Rv2585c*	Unknown	
3,296,843 (A>G)	*Rv2947c (pks15)*	Polyketide synthesis	
3,591,063 (T>C)	*Rv3213c*	Unknown (partitioning regulation)	
3,736,628 (T>G)	*Rv3343c (PPE54)*	Unknown	
3,746,409 (A>G)	*Rv3347c (PPE55)*	Unknown	
3,826,684 (C>T)	*Rv3408 (vapC47)*	Unknown	
3,959,418 (C>T)	*Rv3522 (ltp4)*	Unknown (lipid metabolism)	
4,302,036 (T>C)	*Rv3827c*	IS1537 transposition	
**Other LIVs specifically found in *Mtb* causing TBM compared to PulTB**			
71,336 (G>C)	*Rv0064A (vapB1)*	Unknown	Upstream gene variant
132,417 (C>G)	*Rv0107c (ctpI)*	Cation-transporting ATPase	Upstream gene variant
837,033 (A>G)	*Rv0728c-Rvnt09 (serA2-thrV)*	L-serine biosynthesis/ tRNA	Intergenic region
1,636,826 (C>A)	*Rv1439c-1562c (Rv1439c-treZ)*	Unknown/ trehalose biosynthesis	Intergenic region

Supplementary Table S1. Annotation of missense SNPs and lineage independent variants commonly found in TBM isolates of *M. tuberculosis* compared to PulTB. Note: LIV= lineage independent variant.

No.	Positions (SNPs)	Size (bp)	Affected genes	Functions
1	368,305 - 368,332	28	*Rv0304c (PPE5)*	Unknown
2	369,563 - 369,583	21	*Rv0304c (PPE5)*	Unknown
3	372,008 - 372,117	110	*Rv0304c (PPE5)*	Unknown
4	598,532 - 598,870	339	*Rv0507 (mmpL2)*	Unknown (fatty acid transport?)
5	848,706 - 849,164	459	*Rv0755c (PPE12)*	Unknown
6	1,021,831 - 1,021,873	43	*Rv0916c (PE7)- Rv0917 (betP)* intergenic region	Unknown/ glycine betaine uptake
7	1,340,761 - 1,340,842	83	*Rv1197 (esxK)*	Unknown
8	1,985,087 - 1,985,110	24	*Rv1754c*	Unknown
9	2,169,553 - 2,169,578	26	*Rv1918c (PPE35)*	Unknown
10	2,616,893 - 2,617,134	242	*Rv2339 (mmpL9)*	Unknown (fatty acid transport?)
11	3,047,645 - 3,047,709	65	*Rv2734 and Rv2735c*	Unknown/ Unknown
12	3,482,733 - 3,482,800	68	*Rv3115 – Rv3116 (moeB)* intergenic region	IS*1081* insertion/ molybdopterin metabolism
13	3,527,891 - 3,528,048	158	*Rv3159c (PPE53)*	Unknown
14	3,798,712 - 3,798,742	31	*Rv3383c (idsB) – Rv3384c (vapC46)* intergenic region	Membrane lipids biosynthesis / Unknown

Supplementary Table S2. Characteristic of large indels identified from genomic positional comparison between *M. tuberculosis* isolates from TBM and PulTB cases.
